# tRFTars: predicting the targets of tRNA-derived fragments

**DOI:** 10.1186/s12967-021-02731-7

**Published:** 2021-02-25

**Authors:** Qiong Xiao, Peng Gao, Xuanzhang Huang, Xiaowan Chen, Quan Chen, Xinger Lv, Yu Fu, Yongxi Song, Zhenning Wang

**Affiliations:** Department of Surgical Oncology and General Surgery, Key Laboratory of Precision Diagnosis and Treatment of Gastrointestinal Tumors, Ministry of Education, The First Affiliated Hospital of China Medical University, 155 North Nanjing Street, Heping District, Shenyang, 110001 China

**Keywords:** tRNA derived fragments, Crosslinking, ligation and sequencing of hybrids, Features of tRF targeting, Support vector machine, The first tRF target predicting tool

## Abstract

**Background:**

tRNA-derived fragments (tRFs) are 14–40-nucleotide-long, small non-coding RNAs derived from specific tRNA cleavage events with key regulatory functions in many biological processes. Many studies have shown that tRFs are associated with Argonaute (AGO) complexes and inhibit gene expression in the same manner as miRNAs. However, there are currently no tools for accurately predicting tRF target genes.

**Methods:**

We used tRF-mRNA pairs identified by crosslinking, ligation, and sequencing of hybrids (CLASH) and covalent ligation of endogenous AGO-bound RNAs (CLEAR)-CLIP to assess features that may participate in tRF targeting, including the sequence context of each site and tRF-mRNA interactions. We applied genetic algorithm (GA) to select key features and support vector machine (SVM) to construct tRF prediction models.

**Results:**

We first identified features that globally influenced tRF targeting. Among these features, the most significant were the minimum free folding energy (MFE), position 8 match, number of bases paired in the tRF-mRNA duplex, and length of the tRF, which were consistent with previous findings. Our constructed model yielded an area under the receiver operating characteristic (ROC) curve (AUC) = 0.980 (0.977–0.983) in the training process and an AUC = 0.847 (0.83–0.861) in the test process. The model was applied to all the sites with perfect Watson–Crick complementarity to the seed in the 3′ untranslated region (3′-UTR) of the human genome. Seven of nine target/nontarget genes of tRFs confirmed by reporter assay were predicted. We also validated the predictions via quantitative real-time PCR (qRT-PCR). Thirteen potential target genes from the top of the predictions were significantly down-regulated at the mRNA levels by overexpression of the tRFs (tRF-3001a, tRF-3003a or tRF-3009a).

**Conclusions:**

Predictions can be obtained online, tRFTars, freely available at http://trftars.cmuzhenninglab.org:3838/tar/, which is the first tool to predict targets of tRFs in humans with a user-friendly interface.

## Background

tRNA-derived fragments (tRFs) are small non-coding RNAs derived from tRNAs with lengths of 14–40 nucleotides (nts). They have been identified at high abundances in many species [1–3] and can be divided into five categories: (i) tRF-5 s, from the 5′ ends of mature tRNAs; (ii) tRF-3 s, from the 3′ ends of mature tRNAs with 3′-CCA termini; (iii) i-tRFs, from the internal cleavage of mature tRNAs; (iv) tRF-1 s (3′U tRFs), from the 3′ trailing sequences of pre-tRNAs with poly-U residues; and (v) tiRNAs, tRNA halves from cleavage at the anticodon of mature tRNAs [2–4]. Accumulating evidence has shown that tRFs are derived from specific tRNA cleavage events catalyzed by enzymes such as angiogenin, tRNase Z, RNase P and Dicer (not necessary) rather than random tRNA degradation [5]. Since databases for identifying and storing tRF sequences such as tRFdb and tRF2Cancer have been published, many studies have reported the roles of tRFs in humans [6–10]. Deep sequence analysis of small RNAs associated with Argonaute (AGO) complexes, the main components of the RNA-induced silencing complex (RISC), has allowed a large number of reads to be mapped to fragments of tRNA [11–13]. Kumar further mined AGO PAR-CLIP data and showed that tRF-3 s and tRF-5 s could associate with target mRNAs by their 5′ seed sequence (tRF nts 2–7) in a manner similar to that of miRNAs [11, 12, 14, 15], with hexamers complementary to the seed referred to as “seed matches”. Moreover, many studies using reporter assays have confirmed that tRFs show activity in regulating the expression of protein-coding genes through complementary pairing between the seed sequence and the 3′ untranslated region (3′-UTR) of the target mRNA [3, 11, 16]. Although seed pairing is commonly thought to function in gene expression regulation [17, 18], studies on specific factors that affect tRF targeting are limited.

Identification of the targets of tRFs is central for characterizing the functional roles of tRFs. However, there have been few investigations of the relationships between tRFs and mRNA, and it is unrealistic to identify all tRF targets by experiments. Researchers have no choice but to rely on algorithms that predict the targets of miRNAs [16, 19, 20]. For example, Maute et al. used TargetScan to predict the targets of tRFGlyGCC, but only one in three was repressed by the tRF [16]. Similarly, Zhang et al. validated only one in five mRNAs predicted by miRanda and RNAhybrid using real-time PCR [21]. The accuracy of such approaches has been poor, and there are currently no better methods for predicting the targets of tRFs in humans. Therefore, a computation tool for predicting the targets of tRFs is urgently needed.

In the present study, we screened features that influenced tRF targeting. Then, we used a support vector machine (SVM) to build models with key features selected by a genetic algorithm (GA) using the pairs identified by crosslinking, ligation, and sequencing of hybrids (CLASH) and covalent ligation of endogenous AGO-bound RNAs (CLEAR)-CLIP to achieve relatively high accuracy in both the training and validation processes. We developed the computational tool tRFTars, available at http://trftars.cmuzhenninglab.org:3838/tar/ (mirror site at http://trftar.cmuzhenninglab.org:3838/tar/), which is the first database for predicting the potential targets of tRFs.

## Methods

### Data preparation and preprocessing

We obtained mRNA sequences from UCSC 2019 [22]. The sequences were annotated according to the human genome (hg19) in RefSeq, and only “NM_” transcripts were retained. tRF-3 and tRF-5 sequences were downloaded from tRFdb [6].

We identified tRF-mRNA pairs in HEK293 cells by CLASH and in Huh-7.5 cells by CLEAR-CLIP [23, 24], which connect AGO-bound small RNAs and target RNAs as chimeric reads in the same RISC complex. After removing adaptors and PCR duplicates, we mapped the reads to the ends of tRNAs as long as possible until mismatches or bulges appeared with blastn (e-value < 0.01) [15]. Only tRFs in tRFdb [6] were selected for further study to avoid including tRNA degradation products. After removing part of the sequence mapped to each tRNA, the remaining fragment of the read was mapped to the hg19 3′-UTR. Only pairs with perfect Watson–Crick complementarities between the tRF seed and 3′-UTR sequence were kept in the positive group (the 3′-UTRs were required to have at least 6 contiguous bases paired with 2–7 bases of the 5′ end of tRFs). After removing the transcripts from the positive group, we searched the remaining 3′-UTRs for segments with perfect seed pairing to the tRFs in CLASH as the background [25], which reflects average levels of the features for all possible seed pairing.

We obtained data from poly(A)-position profiling by sequencing (3P-seq) with TargetScan 7.2 [26, 27], which measured 3′-UTR isoform quantifications. When multiple 3′-UTRs mapped to the same genomic region, those with the most 3P-seq tags in the corresponding cell line were chosen. The pipeline of the process is presented in Additional file 1: Figure S1.

### Computational features

After identifying the interactions as described above, we analyzed the features of the positive pairs in CLASH and the background. We mainly focused on potential target sites with seed pairing and considered the pairing type, location, and base identity around them. Then, we assessed the sequence properties of the whole transcripts and tRFs involved in pairing to view their effects on the target sites. Moreover, we selected the local 3′-UTR regions of the seed matches and considered their individual secondary structures and stabilities after binding to tRFs. The collected information is listed in Additional file 1: Table S1.

Notably, when considering the effect of base components near the target site, we assumed that the identity of bases surrounding the seed matches with different distances had different weights. The score for AU bases was computed following the rubric below [28]:

$${\text{S}}_{{{\text{AU}}}} \; = \;\sum\limits_{{{\text{i}} = 1}}^{{{\text{i}} = {\text{n}}}} 1 /{\text{di}}_{{{\text{A/U}}}}  $$

where di _A/U_ denotes the distance of an A or a U to the seed match within a particular range. We separately computed scores of 35 nts upstream or 15 nts downstream of the site. Considering more bases tended to pair with tRF beyond seed pairing, we therefore also computed the score excluding 10 nts immediately upstream of the seed match.

The main secondary structure of the target region was computed by the “RNAfold” program in the ViennaRNA 2.0 package (Lorenz et al. 2011, http://www.tbi.univie.ac.at/RNA) [29] with sequences including 40 nts upstream and 40 nts downstream of the seed match. The minimum free folding energy (MFE) of the duplex was computed by the “RNAup” program in the ViennaRNA 2.0 package (Lorenz et al. 2011, http://www.tbi.univie.ac.at/RNA). All features listed were compared between the positive group and the background.

### The model constructed by the GA and SVM

We used SVM to construct a model for predicting the targets of tRFs. To balance the numbers of pairs in the positive and negative groups during training, we randomly chose 2000 of the tRF-mRNA pairs with the most 3P-seq tags (five times the number in the positive group) from the background as the negative group. To reduce the risks of overfitting and selection bias and to facilitate parameter optimization, fivefold cross-validation was conducted to build the model. All the pairs (pairs identified by CLASH or CLEAR-CLIP and negative pairs) were randomly divided into five subsamples of equal size, with four subsamples used for training and the remaining subsample used for testing each time. This process was repeated five times, with each subsample tested exactly once. To improve training efficiency and retain appropriate features, the features mentioned above were selected by GA reflecting the process of natural selection. We set the number of iterations of the GA to 10,000, with possibilities of crossing and mutation of 10% and 30%, respectively. Finally, we averaged the positive probabilities of individual target sites for each fold. We assumed that multiple sites typically acted independently from each other. The positive probability for each potential target transcript with seed matches was computed as follows:

$${\text{P}}\; = \;1\; - \;\prod\limits_{{{\text{i}} = 1}}^{{\text{n}}} {{\text{pi}}}  $$

where n denotes the number of complementary sites in the transcripts and pi denotes the possibility of an individual site being predicted as functional by the SVM model. The pairs with a positive probability > 0.5 were selected as targets of a specific tRF.

### The probabilistic model for potential target transcripts

We computed the probability of a specific seed match appearing at a potential 3′-UTR target as P by Markov model (MM) (order 1), depending on the base composition of the sequence. For a specific tRF-3′-UTR pair, we performed a binomial test to compute ${\text{Ps}}$ as follows:

$$ {\text{Ps}} = \mathop \sum \limits_{{{\text{i}} = {\text{f}}}}^{{{\text{l}} - {\text{k}} + 1}} \left( {\begin{array}{*{20}c} {{\text{l}} - {\text{k}} + 1} \\ {\text{i}} \\ \end{array} } \right){\text{P}}^{{\text{i}}} \left( {1 - {\text{P}}} \right)^{{{\text{l}} - {\text{k}} + 1 - {\text{i}}}} $$

where l represents the length of the target sequence, k represents the number of bases in the seed (k = 6), and f represents the frequency of seed matches in the potential target transcripts. We used this formula to calculate ${\text{Ps}}$ for all tRF-3′-UTR pairs, adjusted the calculated ${\text{Ps}}$ values using the Benjamini–Hochberg procedure and evaluated the false discovery rate (FDR).

### Conservation of the target sites

To support our analysis, we used the Bioconductor package GenomicScores, which conveniently provided genome-wide position-specific scores [30]. By loading a Bioconductor annotation package (phastCons100way.UCSC.hg19), we obtained phastCons conservation scores, which were based on a two-state phylogenetic hidden MM (phylo-HMM) and multiple alignments of the human genome (hg19) to the genomes of 99 other vertebrate species [31]. The scores of each seed region, 35 nts upstream, 15 nts downstream, and the whole 3′-UTR were compared between the target and background.

### Database organization and web interface

**Fig. 1:**
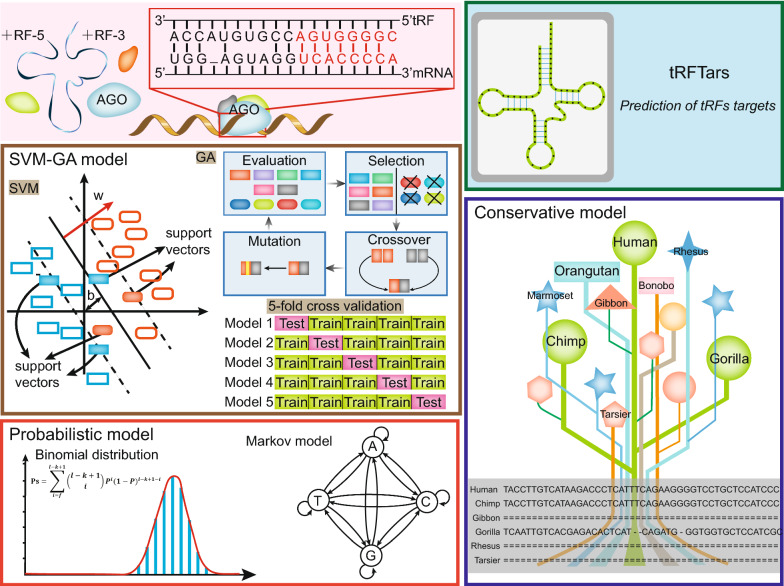
The workflow of the target prediction pipeline of tRFTars

The tRF target prediction workflow is shown in Fig. 1. tRFTars was implemented by the R package Shiny and hosted on a Linux server (Centos 7.5) with MySQL 5.7.18 as its database engine. The web layout was built by the R package shinydashboard, with results shown in interactive tables by the R package DT.

### Algorithm evaluation and comparison with miRNA target prediction models

To evaluate the universal applicability of tRFTars, the SVM-GA model was compared with the commonly used miRNA target predicting algorithms TargetScan [27] and miRanda [32] with the default parameters. The conservative model and the probabilistic model were also considered as independent prediction criteria to be tested. We compared the receiver operation characteristic (ROC) curves of these methods in predicting pairs from CLASH/CLEAR-CLIP. Moreover, the sensitivity, specificity and Matthews correlation coefficient (MCC) of various methods were assessed using the following formulas [33]:

$$ {\text{Sensitivity}}\; = \;\frac{{{\text{TP}}}}{{{\text{TP}} + {\text{FN}}}} $$

$$ {\text{Specificity}}\;{ = }\;\frac{{{\text{TN}}}}{{{\text{TN}} + {\text{FP}}}} $$

$$ {\text{MCC}}\; = \;\frac{{{\text{TP*TN}} - {\text{FP*FN}}}}{{\sqrt {\left( {{\text{TP}} + {\text{FP}}} \right)\left( {{\text{TP}} + {\text{FN}}} \right)\left( {{\text{TN}} + {\text{FP}}} \right)\left( {{\text{TN}} + {\text{FN}}} \right)} }} $$

where TP, TN, FP and FN denote the numbers of true positives, true negatives, false positives and false negatives, respectively, which reflect the consistency of prediction and the experimental results. MCC values range between − 1 and 1, indicating the correlations between predictions and experimental observations. The experimentally tested pairs identified in the reporter assay were employed to further evaluate the performance of our models.

### Validation of prediction results with expression profiles

We quantified the expression of tRFs, miRNAs and mRNAs in 20 gastric tumors and matched adjacent normal tissues from our institution. The steps of sample preparation, sequencing and microarray analysis are presented in the Additional file 1: Methods. We calculated the Spearman correlation coefficient between mRNA and tRF/miRNAs expression levels. All the pairs with seed matches and negative expression correlation coefficients were selected for further analysis. We defined the pairs with correlation coefficients < − 0.3 as negatively related pairs [34]. Moreover, the negatively related tRF-mRNA pairs with seed matches in TCGA database were downloaded [34]. Considering the complexity of expression-related regulation in tissues, we chose only the tRFs in tRFdb with CLASH reads. The most relevant pairs in each cancer with correlation coefficients < − 0.3 in TCGA (P < 5E−06) were retained. To eliminate the influence of the total number of target genes predicted by the different tools due to the different cut-offs, we used a chi-square test to calculate whether the predicted targets of the SVM-GA model were more likely to be downregulated and to compare our predictions with the results from TargetScan and miRanda.

We selected potential target genes of tRF-3001a, tRF-3003a and tRF-3009a from the top of the predictions. We measured the potential target gene expression levels after transfection the tRFs through quantitative real-time PCR (qRT-PCR) in MGC-803 cells. We mutated part of the tRFs seed sequence to the complementary form and observed the expression of the potential target genes (Additional file 1: Methods).

### Statistical programs and software

Statistical analyses of our results were conducted with R version 3.5.3 (https://www.r-project.org/). Statistical significance (P-value) for the features between positive group and the background was calculated with Student’s t-test and a threshold value of P < 0.05 was considered statistically significant (with exceptional circumstances explained individually). The SVM algorithm was built with the LIBSVM program (Chang et al. 2018, https://www.csie.ntu.edu.tw/~cjlin/libsvm/). The SVM and GA algorithm was coded with MATLAB 2016a (MathWorks, Natick, MA, USA). The source code is freely available at Github (https://github.com/cmuxiaoqiong/SVM_GA_tRF_targets).

## Results

### Identification of tRF targets from CLASH data

Based on CLASH data from HEK293 cells and CLEAR-CLIP data from Huh-7.5 cells, we obtained 547 tRF-mRNA pairs (532 from CLASH and 15 from CLEAR-CLIP) involving 28 tRFs (20 tRF-3 s and eight tRF-5 s) in CLASH and 15 tRFs (six tRF-3 s and nine tRF-5 s) in CLEAR-CLIP. A total of 115,418 seed matches paired with the tRFs were used as the background.

### Characterizing target recognition features

**Fig. 2:**
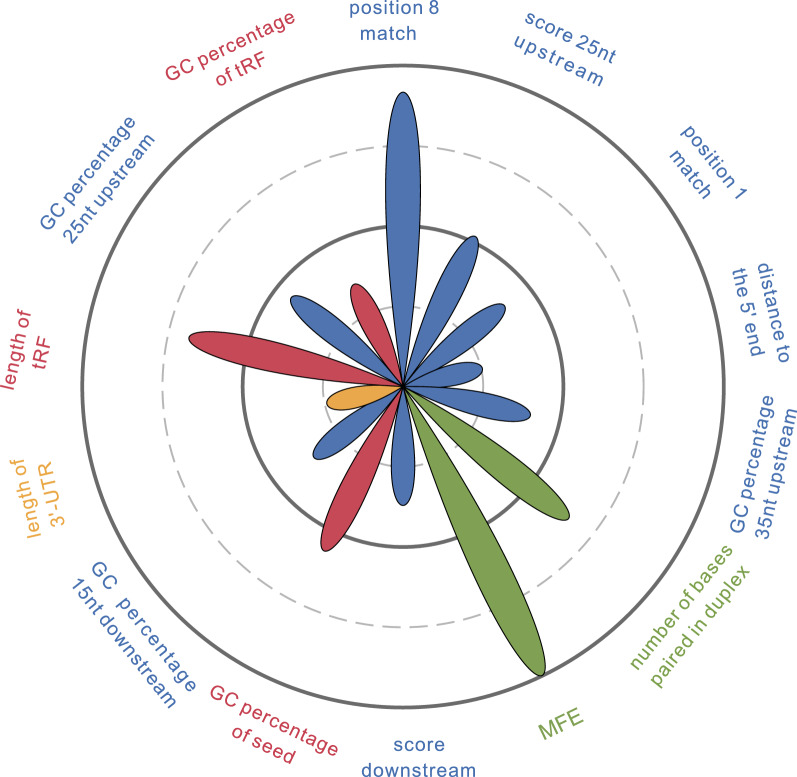
Features that influence tRF targeting. Features most significantly different between the positive group and background are displayed. The size of the petal represents -log(P value), which indicates the degree of significance

The P-values of features considered in this study that may be related to tRF-mRNA interactions are listed in Table 1. We observed significant differences in most sequence features of individual seed matches, transcripts, or tRFs and the features of the tRF-mRNA duplex. The features most significantly different between the positive group and background are shown in Fig. 2.

**Table 1:** The P-value of features considered in this study that can contribute to tRF-mRNA interaction

Category	Feature	Including feature	P value
Sequence feature of target sites	Type of seed match	Position 8 match	2.87E−28
		Position 1 match	7.28E−13
		Position 1 A	0.34
	Bases identity immediately flanking seed match (2nt)	Base identity of position 2 upstream	0.80
		Base identity of position 1 upstream	2.88E−03
		Base identity of position 1 downstream	7.67E−07
		Base identity of position 2 downstream	0.43
	Bases component in the vicinity of seed match	GC percentage 35 nt upstream	5.89E−13
		GC percentage 25 nt upstream(excluding 10 nts immediately upstream)	4.28E−14
		GC percentage 15 nt downstream	2.18E−11
		Score 35 nt upstream	7.51E−03
		Score 25 nt Upstream (excluding 10 nts immediately upstream)	2.61E−16
		Score downstream	7.49E−12
		Base component 35 nt upstream	See Additional files 1, 2, 3: Table
		Base component 15 nt downstream	See Additional file 1, 2, 3: Table
		Dinucleotide component 35 nt upstream	See Additional file 1, 2, 3: Table
		Dinucleotide component 15 nt downstream	See Additional file 1, 2, 3: Table
	Distance to the end of tRF	Distance to the 5′ end	2.37E−08
		Distance to the 3′ end	1.90E−03
		Distance to the nearest end	2.12E−03
Sequence feature of transcripts	3′-UTR properties	Length of 3′-UTR	4.80E−08
		GC percentage of 3′-UTR	5.40E−06
		frequency of seed matches in 3′-UTR	0.08
		Base component(AGCT) of 3′-UTR	See Additional file 1, 2, 3: Table
		Dinucleotide component of 3′-UTR	See Additional file 1, 2, 3: Table
	CDS properties	Length of CDS	1.95E−04
		GC percentage of CDS	2.33E−03
		Frequency of seed matches in CDS	6.51E−06
	5′-UTR properties	length of 5′-UTR	0.40
		GC percentage of 5′-UTR	0.08
		Frequency of seed matches in 5′-UTR	0.02
Sequence feature of tRFs	tRF sequence properties	Length of tRF	2.77E−21
		GC percentage of tRF	2.46E−11
	TA	Target abundance in genome	7.19E−03
		Target abundance in 3′-UTR	0.09
	SPS	GC percentage of seed	8.69E−18
Stability and thermodynamics of tRF-mRNA interaction	Secondary structure of target mRNA	Nucleotides exposed at seed match	7.49E−03
		Nucleotides exposed surrounding seed match	0.99
		Energy to free base-pairing interactions of target site	3.51E−06
	Secondary structure of tRF-mRNA	Number of bases paired in duplex	1.53E−20
		MFE	1.36E−57

#### Sequence features of target sites

Seed match types and surrounding base properties determined the potential of a tRF target site. Position 8 matches (P = 2.87E−28) and position 1 matches (P = 7.28E−13) were more common in the positive group than in the background, while no significant difference was observed at position 1 A (P = 0.34). The number of different kinds of seed types is provided in Additional file 1: Table S2. Consistent with the finding of PAR-CLIP [14, 15], 7-mer-m8 sites (binding to positions 2–8 in the tRFs) were most enriched in the positive group. The bases flanking the functional sites were more likely to be A or U compared with the bases in the background. The differences in nucleotide identity are listed in Additional file 1: Table S3 and Additional file 1: Table S4. The GC percentages were significantly lower for bases upstream (P = 5.89E-13) and downstream (P = 2.18E−11). When using the formula to incorporate distances to seed matches with different weights, the scores in the positive group were significantly higher for all three kinds of surrounding regions, including upstream (P = 7.51E−03), upstream excluding 10 nts (P = 2.61E−16), and downstream (P = 7.49E−12).

The locations of seed matches in the 3′-UTRs were related to the tRF binding activity of the UTRs. The cumulative distribution curves of the distance to the 3′-UTR ends are shown in Additional file 1: Figure S2. We observed that effective sites avoided appearing immediately downstream of the stop codon, while tended to reside adjacent to the ends within the rest of the 3′-UTR in the positive group (P = 2.12E−03), especially the 5′ end (P = 2.37E−08).

#### Sequence features of the transcripts

When focusing on the whole-sequence features to assess their impacts on the efficacies of sites, we found that the positive group had a significantly lower global GC content than the background in the 3′-UTR (P = 5.40E−06), but this trend was not as significant as that detected in local comparisons near seed matches. In agreement with the finding that more sites were preferentially adjacent to the ends, the target sites were selectively located in the shorter 3′-UTRs (P = 4.80E−08).

#### Sequence features of tRFs

We investigated whether some tRF sequences might be intrinsically more capable of targeting than others. In tRFdb, tRF-3 and tRF-5 sequences could be categorized into tRF-3a (18 nts), tRF-3b (22 nts), tRF-5a (15 nts), tRF-5b (22 nts), and tRF-5c (31 nts) sequences according to their lengths. Compared to those in the background, we observed significant differences in the lengths of tRFs (lengths in tRFdb) (P = 2.77E−21), consistent with the finding that repression was mediated by tRF-3as instead of tRF-3bs derived from the 3′ end of the same tRNA [11]. tRFs in the positive group had a higher GC percentage for both whole sequences (P = 2.46E−11) and seed sites (P = 8.69E−18), which could contribute to pairing stability, especially for seed regions [35]. In addition, we detected significant differences in target site abundance (TA) in the genome (P = 7.19E−03), consistent with the finding that extensive pairing could decrease the function of sRNA pairing to their authentic target sites [36–38].

#### Structure and thermodynamic properties of the tRF-mRNA duplex

In addition to the sequence features of tRFs and their targets, the secondary structures of target mRNAs likely contribute to target recognition. We showed that nucleotides in the positive group were more exposed than those in the background at the seed matches (P = 7.49E−03), while less energy was needed to free base-pairing interactions within the secondary structures of target mRNAs (P = 3.51E−06). These features indicated that effective target sites were more accessible for tRF binding.

Thermodynamic features of the tRF-mRNA duplex were then analyzed. Compared with that in the background, the tRF seed-target binding in the positive group was usually more stable, as revealed by a lower MFE (P = 1.36E−57), which represented the most stable structure of the helix. Moreover, additional pairing beyond seed matches contributed to target recognition in the positive group (P = 1.53E−20), consistent with the experimental discovery of Kuscu et al. [11]. These features were considered in our models.

### The target prediction model established by SVM and GA

**Fig. 3:**
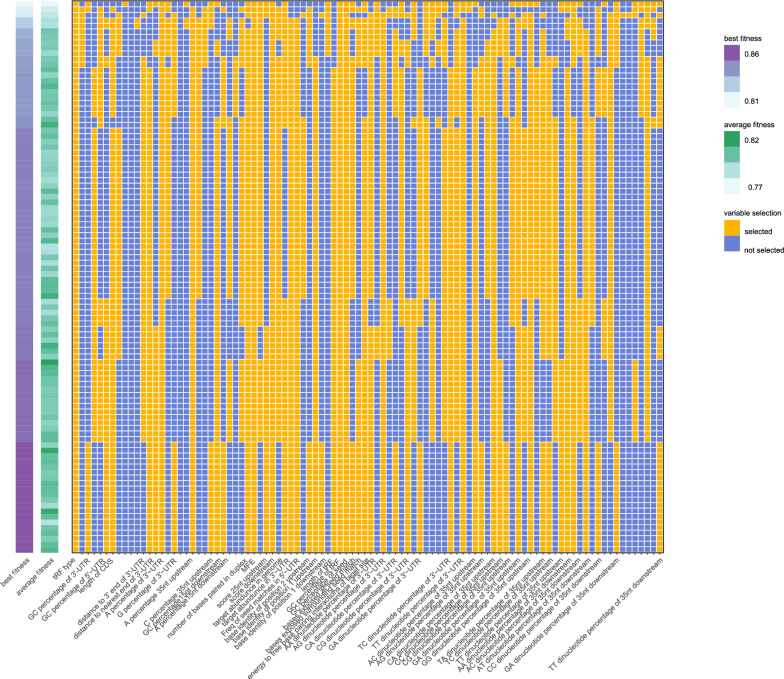
The result of the genetic algorithm (GA) during establishment of the SVM-GA model using the training cohort. The iterations of each variable in the GA are presented in the longitudinal axes and the selected variables in the SVM-GA model are presented in the transverse axes. The features selected are labeled

A total of 547 positive pairs (including 489 tRF-3 pairs and 58 tRF-5 pairs) and 2000 negative pairs (including 1596 tRF-3 pairs and 404 tRF-5 pairs) were retained according to our filtering criteria. The features with significant differences between the positive group and negative group are listed in Additional file 1: Table S5, with no significant differences observed for the vast majority of features between CLASH and CLEAR-CLIP. We considered the 96 features listed in Table 1 as potentially informative in relation to tRF targeting, while 51 features were identified by the GA after parameter optimization (Fig. 3). These features were modeled in an SVM framework to determine their individual contributions for model implementation. The result of each fold was stable, with an area under the ROC curve (AUC) = 0.980 (from 0.977 to 0.983) during the training process and an AUC = 0.847 (from 0.83 to 0.861) during the validation process (Additional file 1: Table S6). Fourteen of 15 pairs detected by CLEAR-CLIP and 455 of 532 detected by CLASH were predicted, which indicated the efficiency of our model in different cell lines.

### Predicting the targets with the probabilistic model

Functional tRF-target interactions, which account for a small proportion of seed pairings, derive directly from coevolution of the tRF and its target. Potential functional 3′-UTR targets should contain more complementary sites overrepresented relative to a random background, which was measured by Ps for each tRF-3′-UTR pair. Ps was significantly lower in the positive group than in the background (P = 2.94E−10). We generated the final predictions by ranking adjusted Ps, showing that 26,380 tRF-3 targets and 8670 tRF-5 targets were overrepresented. This method could be used to independently predict the targets of tRFs by simulating miRNA target prediction tools such as PicTar and PACMIT [39, 40].

### Predicting the targets with conserved seed match properties

Biologically functional target sites tend to be located in conserved tRF-pairing motifs within 3′-UTRs [18]. We found that target sites were significantly more conserved than the background (P = 3.68E−08), as determined by phastCons scores from comparative sequence analyses of the human genome (hg19) with the genomes of 99 other vertebrate species. Similar levels of performance were observed immediately upstream (P = 2.31E−10), immediately downstream (P = 8.29E−10), and in the whole transcript (P = 3.66E−13). We applied this measure of performance as an independent factor to predict the targets of tRFs [25, 31, 41]. A total of 111,874 tRF-3 target sites and 152,464 tRF-5 target sites were predicted with 0.5 as a cut-off. We performed gene ontology enrichment analysis on the target genes predicted by the conservation analysis. The results were listed in Additional file 2: Table S7.

### Algorithm evaluation of tRF target prediction models

**Fig. 4:**
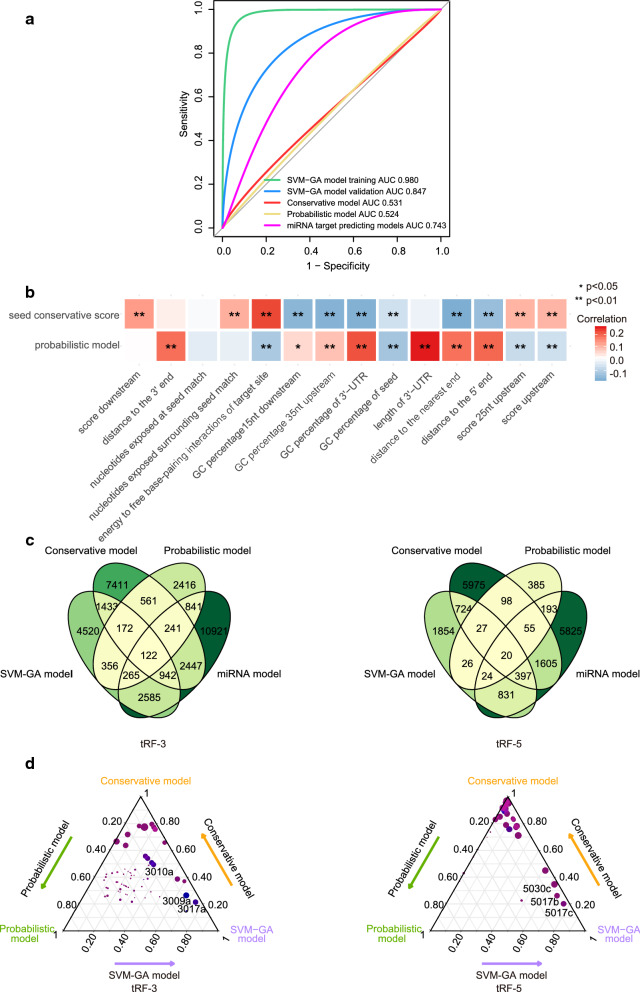
Comparison of tRF target predicting models. **a** The receiver operating characteristic (ROC) curve for classification of the pairs for model establishment, including SVM-GA model, conservation model, probabilistic model and intersection of miRNA target predicting models (TargetScan and miRanda). **b** The relationship of features for potential target site with the probabilistic model or conservative model. The color of boxes represents the coefficient of correlations and *represents the significance of correlations (Pearson’s test). **c** The intersection of target genes (tRFs in CLASH) by three methods of tRFTars and miRNA target predicting models (TargetScan and miRanda). **d** Ternary plot of the number of targets of each tRFs. The value to each axis represents the proportion of targets predicted by corresponding models relative to all potential targets. The node color represents the number of potential targets by intersection of three models. As the number of targets increased, the node color changes from red to blue. The node size indicates the number of seed pairings in whole 3′-UTR. The larger the node is, the greater number of seed matches the 3′-UTRs have

The prediction abilities of tRFTars and common miRNA target prediction programs were assessed by comprehensively comparing their identified pairs with the pairs identified by CLASH/CLEAR-CLIP or reporter assays. The performance of our model was evaluated with ROC curve analysis, yielding an AUC = 0.980 in the training process and an AUC = 0.847 in the validation process (Fig. 4a), better than commonly used miRNA target prediction models (intersection of TargetScan and miRanda) (AUC = 0.743, P < 0.0001). Moreover, five of seven positive pairs and two of two negative pairs were predicted by the SVM-GA model (Table 2) [11, 16, 42], while only three of seven positive pairs were predicted by miRNA models. The sensitivity, specificity and MCC are listed in Additional file 1: Table S8. Both lines of evidence suggested that the SVM-GA model was the most effective tool for distinguishing the targets of tRFs with a relatively high accuracy. In addition, we searched the pairs confirmed by reporter assay with a clear tRF sequence beyond those in tRFdb, and KLF12 was predicted to be the target of tRFGluTTC (positive probability = 0.76) [43], which further proved the effectiveness of our model.

**Table 2:** Validation of the models with tRF-mRNA pairs reported by reporter assay

tRF	Gene	Whether target of tRF	References	Prediction of SVM-GA model	Prediction of conservative model	Prediction of probabilistic model	Prediction of miRNA model
3027b	RPA1	Yes	Maute et al. [16]	0.58 (Yes)	0 (No)	0.06 (No)	Yes
3009a	DGCR2	Yes	Kuscu et al. [11]	0.26 (No)	0 (No)	0.72 (No)	No
3009a	SLC6A9	Yes	Kuscu et al. [11]	0.89 (Yes)	0 (No)	0.30 (No)	No
3009a	SMAD1	Yes	Kuscu et al. [11]	0.73 (Yes)	0.62 (Yes)	0.03 (No)	No
3009a	TBL1X	Yes	Kuscu et al. [11]	0.10 (No)	0 (No)	0.32 (No)	No
3009a	FER	Yes	Kuscu et al. [11]	0.86 (Yes)	0 (No)	0.05 (No)	Yes
5030c	LRP8	Yes	Deng et al. [42]	0.89 (Yes)	0.47 (No)	0.17 (No)	Yes
3027b	STAG2	No	Maute et al. [16]	0.22 (No)	1 (Yes)	0.01 (No)	No
3027b	NSD3	No	Maute et al. [16]	0.21 (No)	1 (Yes)	0.02 (No)	No

Moreover, we investigated the relationships between different tRF target prediction models. We discovered that most of the features for effective target site prediction were correlated with the probabilistic model or conservative model (Fig. 4b). The intersections of three algorithms and miRNA target prediction models (tRFs found in CLASH) are displayed in Fig. 4c, and the intersections are listed in Additional file 1: Table S9. The proportion of targets predicted by each model is presented in Fig. 4d.

### Validation of prediction results with expression correlations

We applied the filtering criteria and selected 1226 tRF-mRNA pairs with negative expression correlations in TCGA database [34]. The pairs predicted by the SVM-GA model were more likely to have negative expression correlations (P = 8.97E−04), and the model performed better than TargetScan (P = 0.79) (Additional file 1: Table S10). We have listed the number of pairs in different kinds of tumors in Additional file 1: Table S11. Moreover, by analyzing tsRNA sequencing (tsRNA-Seq) and lncRNA + mRNA microarray data from our institution, 34 tRFs with adequate variability and their expression-related transcripts were chosen. Compared with those of TargetScan and miRanda, the predictions of the SVM-GA model were more enriched in pairs with negative correlations (SVM-GA model: P = 3.09E−03; TargetScan and miRanda: P = 0.09) (Additional file 1: Table S12). Additionally, we observed significant consistency between the correlation coefficients of the pairs and the likelihood of a positive result from the SVM-GA model (for tRF-5s: P = 1.87E−10; for tRF-3s: P = 2.77E−05). This finding indicates that our predicted tRF targets with higher scores tend to be more downregulated. We used Multiple Linear Regression to exclude the influence of miRNA in the sequencing and microarray data from our institution. We chose the pairs with mRNAs regulated by multiple highly expressed miRNAs, and 96 of 397 tRF-mRNA pairs still had significant negative correlation, listed at Additional file 3: Table S13. It showed that nearly 1/4 tRF-mRNA pairs played a most vital role in gene expression regulation. We showed the correlation heatmap of 20 tRFs/miRNAs and mRNAs with the highest frequency among the target gene predictions (Additional file 1: Figure S3).

### Validation the predictions by qRT-PCR

We selected potential target genes of tRF-3001a (ELAVL1, SOCS7, ATF6B, RINL, PRR11, and ZNF268), potential target genes of tRF-3003a (CBX5, EIF4E, PRKAA1, TFDP2, SH3TC2, and PDE12) and potential target genes of tRF-3009a (ATF6B, ARF3, CDS2, CLN8, MAP2K7, and SNX12) from the top of the predictions for further confirmation using qRT-PCR (Additional file 1: Methods). We evaluated potential target gene expression levels after transfection with tRF mimic or the mutated tRF mimic in MGC-803 cells. The expression of 13 target genes was markedly reduced at the mRNA levels by transfecting tRF-mimic compared to the corresponding NC group in MGC-803 cells (Additional file 1: Table S14, Figure S4).

### Website

**Fig. 5:**
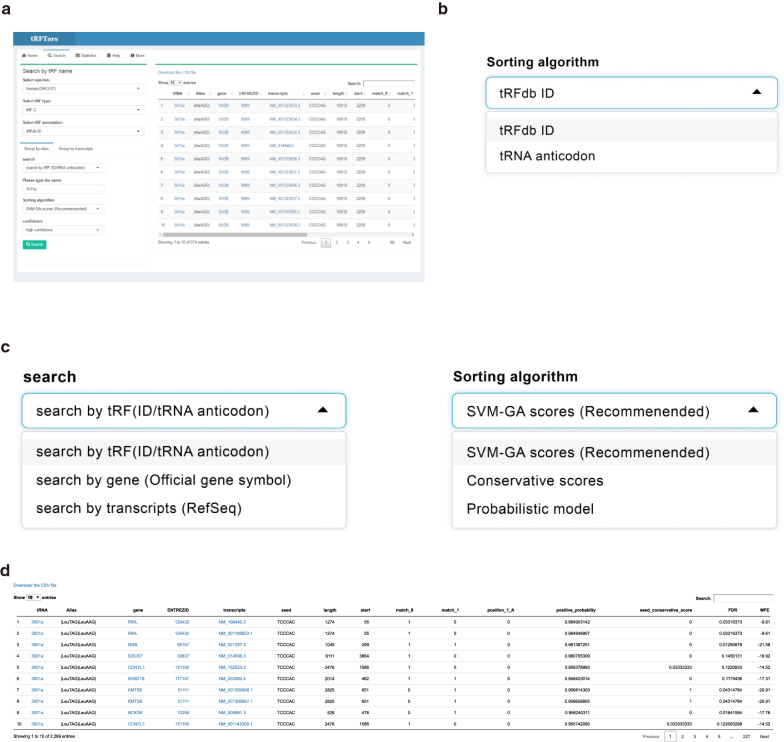
Search the tRF targets in tRFTars with an example of 3001a. **a** Overview of the Search page interface. **b** Users can search the target according to the tRFs in tRFdb or anticodon/ amino acid of the source tRNA. **c** Users can input the corresponding name and rank the target candidates according to positive possibility assigned by the SVM-GA model, the conservative score or the sites overrepresented by the probabilistic model. **d** The result panel of the target site of tRF 3001a

The predicted targets are available online from tRFTars (http://trftars.cmuzhenninglab.org:3838/tar/) (mirror site at http://trftar.cmuzhenninglab.org:3838/tar/) (Fig. 5). Strict matches to the official gene symbols, RefSeq IDs, tRFs in tRFdb or anticodon/amino acids of the source tRNA are necessary as input. Users can choose to search the specified target sites or transcripts according to their needs. Candidates can be ranked according to the likelihood of a positive result assigned by the SVM-GA model, the conservation score or Ps by the probabilistic model, although we recommend adopting the SVM-GA model. Users can choose to comprehensively view the information for all seed pairings. Details of the prediction results can be found on the statistics page of the website.

## Discussion

In this study, we analyzed the results of experiments in which tRFs were ligated to target RNA in purified AGO complexes (CLASH in HEK293 cells and CLEAR-CLIP in Huh-7.5 cells) to identify tRF-mRNA interactions and extended the discovery to all tRFs. We first comprehensively assessed features for effective tRF-mRNA interaction by statistical analysis and discovered that tRFs could indeed act similarly to miRNAs, with most features involved in miRNA targeting significantly different from those in the background. Features with the most significant variance between the positive group and background were the MFE, position 8 match, number of bases paired in the tRF-mRNA duplex, and length of the tRF, which were consistent with previous findings [11, 14]. The intrinsic mechanisms that influenced the interactions can be explained by binding affinity and target site accessibility [28]. SVM was used to incorporate all these features and to determine the contributions of individual features to target predictions.

We validated the effectiveness of our SVM-GA model, obtaining an AUC = 0.847 during the internal validation process. Furthermore, seven of nine target/nontarget genes confirmed by reporter assay were predicted successfully, far greater than the number obtained with miRNA target prediction algorithms. For example, Maute et al. demonstrated that 3027b (CU1276) (human) overexpression by both tRNA and tRF hairpin transfections significantly repressed RPA1 in a B-cell lymphoma line, as determined using 3′-UTR reporter assays, western blotting, and qRT-PCR [16]. NSD3 (WHSC1L1) and STAG2, predicted by TargetScan, showed almost no response to 3027b overexpression. This is consistent with our predictions. Similarly, SMAD1, SLC6A9, and FER (FER1, matching the 3′-UTR sequence of FER in the NCBI database), which were validated to be targets of 3009a (human) using the luciferase reporter assay and RNA-seq by Kuscu et al., were also predicted. Conversely, although Maute et al. found that TargetScan-predicted genes were significantly enriched in downregulated genes by 3027b, the accuracy rate in the study was less than 10% for both tRNA and hairpin transfections [16]. Although this was not a gold standard method, the prediction accuracy was still unsatisfying.

It is undeniable that this is an unfair comparison because these interactions were chosen for validation after prediction with algorithms for miRNAs. However, our SVM-GA model still outperformed the intersection of miRNA target prediction models. We aimed to understand the mechanism underlying this accuracy difference. Though our model and other miRNA target prediction algorithms considered sequence complementarity, thermostatic calculations of duplex formation, and evolutionary properties to rank the potential target candidates, the detailed contributions of individual features were different. For example, different from the relatively fixed length of 22 nts of miRNAs, tRFs have a variable length from 14 to 40 nts. They may act differently from miRNAs, whose 13–16 bases play an essential role in targeting in addition to seed pairing. Moreover, conservation may not be a determinant of tRF targeting. Prediction by a probabilistic model and conservative model will miss some cases because of relatively low sensitivity. While, additional complementarity downstream from the seed sequence is a more essential feature for tRF targeting. Accounting for such complementarity can lead to relatively good model performance by considering the secondary structure of the tRF-mRNA duplex.

However, we investigated why the system missed predicting targets of 3009a, TBL1X and DGCR2 in two cases. Both target sequences were located in the middle of long 3′-UTRs with a relatively high GC content near the seed matches, which was in conflict with the requirements for site accessibility. Although TBL1X had seven additional bases paired with 3009a, it was not the most stable structure because of intrinsic pairing within secondary structures near the seed match, as predicted by RNAup. However, we believe that extensive base pairing can occur when the interacting molecules are present at high concentrations, which could contribute to effective 3009a-TBL1X interactions. Furthermore, although DGCR2 had five additional bases paired with 3009a, the resulting duplex had a higher MFE, which was contrary to the expectation of site affinity.

This study had some limitations. First, we did not allow any mismatches or bulges when mapping reads to tRNAs or 3′-UTRs. To avoid influences of the RNase step in the CLASH protocol and the inclusion of tRFs from random tRNA degradation products, we included only the tRFs in tRFdb in our model. We adopted stringent inclusion criteria at the expense of excluding some effective pairs in the positive group. Second, it was difficult to conduct large-scale precision validation of tRF target genes. We observed a tendency for predicted targets with higher positive probability in SVM-GA model to be more suppressed in mRNA levels. More experiments that quantify tRF repression strength by transfection or knockdown of a particular tRF will be conducted in different cells and under different conditions to improve our model. Third, the hg38 sequence information of tRFs is not updated in public databases. Additionally, tiRNAs and tRFs not included in tRFdb, such as 5′-tiRNAVal and tRF5-Glu, which have been reported to repress mRNA by seed pairing [18, 44, 45], were not considered in this version. Fourth, exceptional cases, such as contiguous pairing of the 3′-end and non-canonical binding, need to be further investigated [42]. We will also investigate whether tRF-specific features act in tRF targeting.

## Conclusions

The tRFTars is the first website to predict the target of tRF in humans. We intend to update predictions of the website as more tRFs identified to be functional, and plan to extend our model to more species. This website provides convenience to identify potential human targets of particular tRFs for experimental confirmation. Furthermore, it will greatly facilitate our understanding of gene regulation and the functions of tRFs.

## Supplementary Information

**Additional file 1: Table S1**. Summary of features analyzed in this study that can contribute to tRF-mRNA interaction. **Table S2**. The number of different kinds of seed type. **Table S3**. Bases component of positive pairs compared with background. **Table S4**. Dinucleotide component of positive pairs compared with background. **Table S5**. P value of features with significant difference between positive group and negative group during training. **Table S6**. AUC of each fold during model establishment. **Table S8**. Comparison of tRFTars and other miRNA target predicting models. **Table S9**. The intersection of targets(SVM-GA model, probabilistic model, conservative model and miRNA target predicting model) for the tRFs (CLASH). **Table S10.** Validation of the predictions with the data of TCGA. **Table S11**. The number of pairs in different tumor types. **Table S12**. Validation of the predictions with the microarray and sequencing data for gastric cancer patients’ tumor tissues and matched non-tumor adjacent tissues from our institution. **Table S14**. The pairs validated by qRT-PCR.** Table S15.** List of sequences used in this study. **Figure S1**. The pipeline of data preparation and SVM-GA model construction. **Figure S2**. Cumulative distribution curve of the distance of seed sites to the 5' end, 3' end and nearest end. We observed that 3'-UTR sites immediately near the stop codon were less effective compared to sites elsewhere in the 3'-UTR. More sites within the remainder of the 3'-UTR tended to reside near the ends of the UTRs, especially the 5' end. **Figure S3**. The correlation heatmap of 20 tRFs/miRNAs and mRNAs with the highest frequency among the target gene predictions.** Figure S4.** Expression levels of predicted target genes were detected in MGC-803 cells after transfection with tRF mimics or mutated tRF mimics by qRT-PCR relative to the NC group.

**Additional file 2: Table S7**. Gene ontology enrichment analysis on the target genes predicted by the conservation analysis.

**Additional file 3: Table S13**. Multiple Linear Regression to exclude the influence of miRNA in the sequencing and microarray data from our institution.

## Data Availability

The datasets analyzed in this study are available in the Gene Expression Omnibus (GEO) database (http://www.ncbi.nlm.nih.gov/geo/) (GES50452, GSE73059). The data and source code of prediction model is freely available at Github (https://github.com/cmuxiaoqiong/SVM_GA_tRF_targets).

## Abbreviations

tRFs: tRNA-derived fragments
CLASH: Crosslinking, ligation, and sequencing of hybrids
GA: Genetic algorithm
SVM: Support vector machine
ROC: Receiver operating characteristic curve
AUC: The area under the receiver operating characteristic curve
RISC: RNA-induced silencing complex
3′-UTR: 3′ Untranslated regions
CLEAR-CLIP: Covalent ligation of endogenous Argonaute-bound RNAs
3P-seq: Poly(A)-position profiling by sequencing
MFE: Minimum free folding energy
MM: Markov model
FDR: False discovery rate
phylo-HMM: Phylogenetic hidden Markov model
MCC: Matthews correlation coefficient
TA: Target-site abundance
GEO: Gene expression omnibus
TCGA: The Cancer Genome Atlas
qRT-PCR: Quantitative real-time PCR
