# Supplementary material for: tRFTars: predicting the targets of tRNA-derived fragments
Source: J Transl Med. 2021 Feb 25;19:88. doi: 10.1186/s12967-021-02731-7 (PMC7908658; doi:10.1186/s12967-021-02731-7)
Supplement: Supplementary file 1 — Additional file 1: Table S1. Summary of features analyzed in this study that can contribute to tRF-mRNA interaction. Table S2. The number of different kinds of seed type. Table S3. Bases component of positive pairs compared with background. Table S4. Dinucleotide component of positive pairs compared with background. Table S5. P value of features with significant difference between positive group and negative group during training. Table S6. AUC of each fold during model establishment. Table S8. Comparison of tRFTars and other miRNA target predicting models. Table S9. The intersection of targets(SVM-GA model, probabilistic model, conservative model and miRNA target predicting model) for the tRFs (CLASH). Table S10. Validation of the predictions with the data of TCGA. Table S11. The number of pairs in different tumor types. Table S12. Validation of the predictions with the microarray and sequencing data for gastric cancer patients’ tumor tissues and matched non-tumor adjacent tissues from our institution. Table S14. The pairs validated by qRT-PCR. Table S15. List of sequences used in this study. Figure S1. The pipeline of data preparation and SVM-GA model construction. Figure S2. Cumulative distribution curve of the distance of seed sites to the 5' end, 3' end and nearest end. We observed that 3'-UTR sites immediately near the stop codon were less effective compared to sites elsewhere in the 3'-UTR. More sites within the remainder of the 3'-UTR tended to reside near the ends of the UTRs, especially the 5' end. Figure S3. The correlation heatmap of 20 tRFs/miRNAs and mRNAs with the highest frequency among the target gene predictions. Figure S4. Expression levels of predicted target genes were detected in MGC-803 cells after transfection with tRF mimics or mutated tRF mimics by qRT-PCR relative to the NC group. [file 12967_2021_2731_MOESM1_ESM.pdf]

Methods

1.1. Tissue specimens and cell lines

Twenty tumors and matched non-tumor adjacent tissues (NATs) were collected from gastric cancer (GC) patients undergoing curative surgery between 2016 and 2017 at the First Affiliated Hospital of China Medical University (Shenyang, China). We obtained the patients' written informed consent based on ethical guidelines. The study was approved by the research ethics committee of the First Affiliated Hospital of China Medical University and complied with the Declaration of Helsinki.

Human GC cell lines MGC-803 were obtained from the Shanghai Institutes for Biological Sciences, China Academy of Science (Shanghai, China). The cells were incubated in RPMI 1640 medium (Invitrogen, Carlsbad, USA) plus 10% fetal bovine serum (FBS) at 37 with 5% CO2 (Thermo, Waltham, USA).

1.2. Cell transfection

Cells were plated at 2×105 per well in six-well plates and transiently transfected by 50nM tRF-3001a/tRF-3003a/tRF-3009a mimic, mutated tRF-3001a/tRF-3003a/ tRF-3009a mimic or mimic negative control (mimic-NC) following the manufacturer's protocol. All sequences are shown in Supplementary Table 15. Cells were used 48 h post-transfection for subsequent experimentation.

1.3. RNA extraction and quantitative realtime PCR (qRT-PCR)

TRIzol reagent (Ambion) was used to extract RNAs from tissues and cells following the manufacturer's protocol. Complementary DNA (cDNA) was synthesized using the Mir-XTM miRNA First-Strand Synthesis Kit (Clontech, California, USA) or PrimeScript RT reagent Kit (Takara). A Light Cycler 480 II Real-Time PCR system (Roche Diagnostics) was used to identify gene expression levels. The qRT-PCR program involved thermocycling at 95 for 5 sec, 60 for 20 sec and 55 for 30 sec, repeated 45 cycles. RNU6B (U6) was used to normalize for tRFs. Custom primers (Sangon Biotech) were used, with sequences shown in Supplementary Table 15.

1.4. tsRNA Sequencing (tsRNA-Seq), miRNA Sequencing and LncRNA + mRNA Microarray quantification

Total RNA from GC patients' tumor tissues and matched-paired NATs was quantified via tsRNA/miRNA Sequencing and LncRNA + mRNA Microarray. We used rtStar tRF& tiRNA Pretreatment Kit protocols (Arraystar, USA), and RNA modifications that may interfere with small RNA-seq library construction were removed before library preparation. Small RNA libraries were qualified and absolutely quantified using Agilent BioAnalyzer 2100 and sequenced on Illumina NextSeq 500 system using NextSeq 500/550 V2 kit (Illumina, USA). RNA quantity and quality were measured by NanoDrop ND-1000. RNA integrity was assessed by standard denaturing agarose gel electrophoresis or Agilent 2100 Bioanalyzer.

Arraystar Human LncRNA + mRNA Microarray v4.0 (8 × 60 K) platform was used for the global profiling of human LncRNAs and protein-coding transcripts. About 40,173 LncRNAs and 20,730 coding transcripts were detected by the microarray. Sample labeling and array hybridization were performed according to the Agilent One-Color Microarray-Based Gene Expression Analysis protocol (Agilent Technology).

1.5. Microarray and Sequencing data analysis

Sequencing quality was examined by FastQC software and trimmed reads (pass Illumina quality filter, trimmed 3-adaptor bases by cutadapt) were aligned to mature-tRNA and pre-tRNA sequence getting from GtRNAdb using NovoAlign software (v2.07.11). The expression profiling of tRFs were calculated based on normalized TPM. The miRNA expression levels were measured and normalized as tag counts per million of total aligned miRNA reads (TPM). For each miRNA sequence-based profile, the number of miRNA sequence reads can be used to estimate expression level of each miRNA. Choosing a different isomiR sequence for measuring miRNA expression can affect the ability to detect differential miRNA expression. We use the most abundant isomiR, the mature miRNA annotated in miRBase and all isomiR (5p or 3p) to calculate the miRNAs expression. Agilent Feature Extraction software (version 11.0.1.1) was used to analyze acquired array images. Quantile normalization and subsequent data processing were performed with using the GeneSpring GX v12.1 software package (Agilent Technologies). After

quantile normalization of the raw data, LncRNAs and mRNAs that at least 10 out of 20 samples had flags in Present or Marginal ("All Targets Value") were chosen for further data analysis.

Table S1. Summary of features analyzed in this study that can contribute to tRF-mRNA interaction

Category	Feature	Including feature
Sequence feature of target sites	type of seed match	position 8 match, position 1 match, position 1 A
	bases identity immediately flanking seed match (2nt)	base identity of position 2 upstream, base identity of position 1 upstream, base identity of position 1 downstream, base identity of position 2 downstream
	bases component in the vicinity of seed match	GC percentage 35nt upstream, GC percentage 15nt downstream, base component(AGCT) 35nt upstream, base component(AGCT) 15nt downstream, score 35nt upstream, score 25nt upstream(excluding 10 nts immediately upstream), score downstream, GC percentage 25nt upstream, dinucleotide component 35nt upstream, dinucleotide component 15nt downstream
	distance to the end of 3’-UTR	distance to the 5’ end, distance to the 3’ end, distance to the nearest end
Sequence feature of transcripts	3’-UTR properties	length of 3'-UTR, GC percentage of 3'-UTR, base component(AGCT) of 3'-UTR, frequency of seed matches in 3’-UTR, dinucleotide component of 3'-UTR
	CDS properties	length of CDS, GC percentage of CDS, frequency of seed matches in CDS
	5’-UTR properties	length of 5'-UTR, GC percentage of 5'-UTR, frequency of seed matches in 5'-UTR
Sequence feature of tRFs	tRF sequence properties	length of tRF, GC percentage of tRF
	TA	target abundance in genome, target abundance in 3'-UTR
	SPS	GC percentage of seed
Stability and thermodynamics of tRF-mRNA interaction	secondary structure of target mRNA	nucleotides exposed at seed match, nucleotides exposed surrounding seed match, energy to free base-pairing interactions of target site
	secondary structure of tRF-mRNA	number of bases paired in duplex, MFE

Table S2. The number of different kinds of seed type.

Seed type	Positive	Background
8mer	89	6588
7mer m8	172	20718
7mer m1	127	22153
7mer-A1	74	21883
6mer	118	53208

8mer: An exact match to positions 1-8 of the mature tRF.
7mer-m8: An exact match to positions 2-8 of the mature tRF (the seed + position 8).
7mer-m1: An exact match to positions 1-7 of the mature tRF (the seed + position 1).
7mer-A1: An exact match to positions 2-7 of the mature tRF (the seed) followed by an 'A'.
6mer: An exact match to positions 2-7 of the mature tRF (the seed).

Table S3. Bases component of positive pairs compared with background.

Base type	Whole 3'-UTR	Upstream seed matches	Downstream seed matches
A	0.12	0.05	5.03E-04
C	1.27E-06	7.36E-20	3.85E-08
G	2.20E-03	0.39	0.04
T	7.40E-11	1.48E-12	7.68E-06

Table S4. Dinucleotide component of positive pairs compared with background.

	Whole 3'-UTR	Upstream seed matches	Downstream seed matches
AA	6.01E-03	0.25	0.10
AC	1.88E-04	0.02	0.57
AG	2.26E-08	0.42	0.22
AT	1.05E-04	2.91E-07	1.88E-09
CA	4.53E-13	4.61E-03	0.91
CC	1.21E-03	1.86E-13	4.75E-06
CG	3.11E-07	2.40E-10	2.79E-05
CT	0.07	8.11E-04	5.16E-05
GA	4.17E-03	0.71	0.73
GC	8.27E-10	1.17E-13	1.01E-05
GG	0.06	0.60	0.20
GT	2.59E-08	5.30E-09	0.12
TA	1.00E-05	2.08E-08	5.25E-08
TC	0.04	0.02	0.98
TG	3.24E-04	1.29E-08	0.51
TT	3.87E-10	3.00E-06	3.33E-03

Table S5. P value of features with significant difference between positive group and negative group during training.

Including feature	Positive/Negative	CLASH/CLEAR-CLIP
position 8 match	1.55E-19	0.06
position 1 match	8.27E-12	0.16
T of the 3’-UTR	7.36E-04	0.88
G of the 3’-UTR	0.04	0.59
C of the 3’-UTR	0.02	0.46
base identity of position 1 upstream	0.01	0.19
base identity of position 1 downstream	2.41E-06	0.55
GC percentage 35nt upstream	1.48E-09	0.84
T percentage 35nt upstream	2.09E-09	0.32
C percentage 35nt upstream	4.81E-10	0.01
GC percentage 25nt upstream(excluding 10 nts immediately upstream)	3.98E-11	0.97
GC percentage 15nt downstream	6.84E-10	0.78
A percentage 15nt downstream	5.53E-04	0.82
T percentage 15nt downstream	6.00E-05	0.61
G percentage 15nt downstream	1.74E-03	0.92
C percentage 15nt downstream	1.34E-04	0.75
score 35nt upstream	0.02	0.92
score 25nt upstream(excluding 10 nts immediately upstream)	2.67E-12	0.26
score downstream	1.19E-11	0.36
distance to the 5’ end	4.44E-08	3.87E-08
distance to the 3’ end	1.58E-26	0.08
length of 3'-UTR	0.93	0.50
GC percentage of 3'-UTR	0.01	0.82
frequency of seed matches in 3’-UTR	0.15	0.27
AG dinucleotide component of 3'-UTR	0.01	0.16
AT dinucleotide component of 3'-UTR	0.01	0.93
CG dinucleotide component of 3'-UTR	4.41E-10	0.65
GC dinucleotide component of 3'-UTR	3.55E-05	0.64
TA dinucleotide component of 3'-UTR	0.02	0.99

TG dinucleotide component of 3'-UTR	4.34E-03	0.24
TT dinucleotide component of 3'-UTR	7.34E-03	0.35
AT dinucleotide component 35nt upstream	4.91E-05	0.07
CC dinucleotide component 35nt upstream	8.68E-08	0.13
CG dinucleotide component 35nt upstream	3.91E-13	0.18
GC dinucleotide component 35nt upstream	2.24E-10	0.82
GT dinucleotide component 35nt upstream	3.94E-05	0.25
TA dinucleotide component 35nt upstream	3.49E-05	0.39
TG dinucleotide component 35nt upstream	4.28E-06	0.54
TT dinucleotide component 35nt upstream	7.12E-05	0.63
AT dinucleotide component 15nt downstream	1.31E-06	0.68
CC dinucleotide component 15nt downstream	1.16E-03	0.25
CG dinucleotide component 15nt downstream	1.84E-06	0.33
CT dinucleotide component 15nt downstream	0.02	0.52
GC dinucleotide component 15nt downstream	4.57E-06	0.34
GG dinucleotide component 15nt downstream	0.02	0.05
TA dinucleotide component 15nt downstream	3.48E-05	0.63
TT dinucleotide component 15nt downstream	0.02	0.76
frequency of seed matches in CDS	3.73E-03	0.59
length of 5'-UTR	0.34	0.47
GC percentage of 5'-UTR	0.18	0.89
frequency of seed matches in 5'-UTR	6.82E-04	0.21
length of tRF	1.20E-14	0.13
GC percentage of tRF	1.58E-17	0.18
target abundance in 3'-UTR	1.81E-04	0.14

GC percentage of seed	2.41E-04	0.32
energy to free base-pairing interactions of target site	2.10E-06	0.38
number of bases paired in duplex	1.72E-22	0.15
MFE	3.13E-47	6.71E-03

Table S6. AUC of each fold during model establishment.

	Fold1	Fold2	Fold3	Fold4	Fold5	Average
Training	0.978	0.98	0.983	0.980	0.977	0.980
Validation	0.860	0.848	0.861	0.837	0.830	0.847

Table S8. Comparison of tRFTars and other miRNA target predicting models.

Predictor	CLASH/CLEAR-CLIP dataset			Experiment verification pairs		
	Sensitivity	Specificity	MCC	Sensitivity	Specificity	MCC
tRFTars	0.857	0.992	0.887	0.714	1	0.598
Conservative model	0.256	0.798	0.023	0.143	0	-0.76
Probabilistic model	0.358	0.673	0.027	0	1	NA
miRNA model	0.485	0.760	0.230	0.429	1	0.378

Table S9. The intersection of targets(SVM-GA model, probabilistic model, conservative model and miRNA target predicting model) for the tRFs (CLASH).

tRFs	The intersection of targets
3001a	PTBP2,PPP1R3F,DBNDD2,DNAL4,DNMT3A,LIMK1,FGF14,NRXN2,ZNF594,ZNF544,DDOST,ZNF135,XPR1,GSTCD,ZNF814
3002a	NDUFA8,ALOX5AP
3003a	CACNA2D2
3004a	SET,CNOT1,DTNA
3006a	LRRTM4
3007a	FOSL1
3009a	AP3B1,POU2F2,KLHDC3,HNRNPUL2,H1F0,PIM1,SLC7A6,TPBGL,HMGA1,ARHGAP32,PIK3R1,KBTBD2,TTBK1,FBXW2,MAP2K7,IGLON5,LRRC4,PPIL1,KCNA1,PNISR,TMSB4X,PHF1,CLN8,PLEKHG1,FAM43A,USP42,ERGIC1,CLDN11
3011a	ZNF146,ZNF75A,TCF7L2,ZNF30,ZNF584,ZFP90,ZNF268,ZNF559-ZNF177,ZNF773,RPS27,SLITRK2
3013a	ELFN2
3014a	FAM114A2,GLYCTK,OXA1L,KCTD20,RASSF3
3019a	AQP4,BTBD3,IQSEC2,CA7,INO80D,PWWP2A
3020a	PHF6,TGIF1,API5
3023a	SUPT6H,POLA1
3024a	TUBB3,MSL2,TENM3,POU2F1,COPZ1,APOLD1,ZNF589
3026a	ZIC3,PIM2
3027a	AGO4,GRB2,PRDM16,MTRNR2L5,MN1,ARPC4,KMT2D,DMPK
3029a	FABP3,PPP2R5E,
3031a	TNRC6B,MAFF,DNAJB2,PIM2
3033a	TRIM71,TSPAN9,GATAD2B,LSM14B,FAM120C,PTPA,ERF,NRXN2,FOSB,ARHGEF12,NAT8L,AHNAK,SOCS7,ZNF592,PRXL2B,LRRC4,HNRNPUL2,LDLRAD2,ACTR2,SNX12
5002a	ANKRD63
5003b	MAML1,PDE7B,NPR3
5005a	KMT2D
5017a	EFNA5
5023a	DDX52
5028a	ZNF426,ZNF780A,SCUBE3,MAN2A1,ZNF589,ZNF763,ZNF559,ZNF773,ZNF37A,ZNF302,ZNF594
5030b	JDP2,ZC3H12B

Table S10. Validation of the predictions with the data of TCGA.

	Number of negatively related pairs	Total numbers	The proportion of negatively related pairs	Chi-square test between the particular algorithms and all pairs with seed matches
Pairs predicted by tRFTars	235	12195	0.0193	P=8.97E-04
Pairs predicted by TargetScan and miRanda	308	19944	0.0154	P=0.79
All pairs with seed matches	1226	80953	0.0151	

Table S11. The number of pairs in different tumor types.

Tumor type	Predictions of tRFTars	Predictions of tRFTars	Number of pairs with negative expression correlation
ACC	4	6	25
BLCA	16	25	91
BRCA	7	2	37
CESC	12	9	44
CHOL	2	0	2
DLBC	10	18	60
ESCA	3	7	23
HNSC	3	13	41
KICH	17	9	40
KIRP	0	0	2
LAML	24	45	172
LGG	2	1	19
LIHC	12	14	55
LUAD	8	17	53
LUSC	1	4	19
MESO	11	22	94
OV	0	1	3
PAAD	15	14	85
PRAD	5	13	40
READ	0	2	3
SARC	68	69	228
SKCM	0	0	2
STAD	3	3	12
TGCT	0	1	9
THCA	0	0	1
THYM	21	35	146
UCEC	1	1	1
UCS	3	1	9
UVM	0	0	5

Abbreviations:

ACC: Adrenocortical carcinoma

BLCA: Bladder Urothelial Carcinoma

BRCA: Breast invasive carcinoma

CESC: Cervical squamous cell carcinoma and endocervical adenocarcinoma

CHOL: Cholangiocarcinoma

COAD: Colon adenocarcinoma

DLBC: Lymphoid Neoplasm Diffuse Large B-cell Lymphoma

ESCA: Esophageal carcinoma

HNSC: Head and Neck squamous cell carcinoma
KICH: Kidney Chromophobe
KIRP: Kidney renal papillary cell carcinoma
LAML: Acute Myeloid Leukemia
LGG: Brain Lower Grade Glioma
LIHC: Liver hepatocellular carcinoma
LUAD: Lung adenocarcinoma
LUSC: Lung squamous cell carcinoma
MESO: Mesothelioma
OV: Ovarian serous cystadenocarcinoma
PAAD: Pancreatic adenocarcinoma
PRAD: Prostate adenocarcinoma
READ: Rectum adenocarcinoma
SARC: Sarcoma
SKCM: Skin Cutaneous Melanoma
STAD: Stomach adenocarcinoma
TGCT: Testicular Germ Cell Tumors
THCA: Thyroid carcinoma
THYM: Thymoma
UCEC: Uterine Corpus Endometrial Carcinoma
UCS: Uterine Carcinosarcoma
UVM: Uveal Melanoma

Table S12. Validation of the predictions with the microarray and sequencing data for gastric cancer patients' tumor tissues and matched non-tumor adjacent tissues from our institution.

	Number of negatively related pairs	Total numbers	The proportion of negatively related pairs	Chi-square test between the particular algorithms and all pairs with seed matches
Pairs predicted by tRFTars	1522	5285	0.288	P=3.09E-03
Pairs predicted by TargetScan and miRanda	2064	7503	0.275	P=0.09
All pairs with seed matches	11557	43994	0.263	

Table S14. The pairs validated by qRT-PCR.

**tRF**	**Gene**	Positive probability	Down-regulated by tRF overexpression(P<0.05)
3001a	ELAVL1	0.986	Yes(P=0.04)
3001a	SOCS7	0.973	Yes(P=6.28E-03)
3001a	ATF6B	0.977	Yes(P=4.24E-03)
3001a	RINL	0.965	No(P=0.77)
3001a	PRR11	0.987	No(P=0.67)
3001a	ZNF268	0.998	No(P=0.27)
3003a	CBX5	0.851	Yes(P=8.69E-03)
3003a	EIF4E	0.873	Yes(P=0.02)
3003a	PRKAA1	0.921	Yes(P=0.04)
3003a	TFDP2	0.931	Yes(P=0.03)
3003a	SH3TC2	0.825	No(P=1)
3003a	PDE12	0.812	No(P=0.33)
3009a	ATF6B	1	Yes(P=0.02)
3009a	ARF3	1	Yes(P=0.01)
3009a	CDS2	1	Yes(P=0.02)
3009a	MAP2K7	1	Yes(P=0.02)
3009a	CLN8	1	Yes(P=0.03)
3009a	SNX12	1	Yes(P=8.31E-03)

Table S15. List of sequences used in this study.

Name	Sequence (5' - 3')
tRF-3001a Forward Primer	ATCCCACCGCTGCCAC
tRF-3003a Forward Primer	TCCGGGTGCCCCCTC
tRF-3009a Forward Primer	ACCCCACTCCTGGTACCA
U6 Forward Primer	GGAACGATACAGAGAAGATTAGC
U6 Reverse Primer	TGGAACGCTTCACGAATTTGCG
CLN8 Forward Primer	TGGTCGCTGGCTTTGTCTTC
CLN8 Reverse Primer	AGAACGGTAAGTGGCATTCAG
ATF6B Forward Primer	CAGCACCTTGTATTCTGGCCT
ATF6B Reverse Primer	CCGTCAAACGGGACATCCT
CDS2 Forward Primer	ATGGAGAGACTGCATCGGACA
CDS2 Reverse Primer	GAGGACCTCCGGGGTATCATC
ARF3 Forward Primer	ATGGGCAATATCTTTGGAAACCT
ARF3 Reverse Primer	TGAACCCAATGGTAGGGATGG
MAP2K7 Forward Primer	CCACGTCATTGCCGTTAAGC
MAP2K7 Reverse Primer	GCACGATGTAGGGGCAGTC
SNX12 Forward Primer	ATGTCGGACACGGCAGTAG
SNX12 Reverse Primer	TTACTTGGCGGCCCGTAAG
ZNF268 Forward Primer	CAGCTTCTATTTGGGTCCCAC
ZNF268 Reverse Primer	ATTCTGCGACTCTTCTGCTTC
PRR11 Forward Primer	GAAGCTGGCTAACATCATCCTG
PRR11 Reverse Primer	CTCTGGGTTATGCAGTTCTGG
ELAVL1 Forward	GGGTGACATCGGGAGAACG

Primer	
ELAVL1 Reverse Primer	CTGAACAGGCTTCGTAACTCAT
RINL Forward Primer	GGAGCAGACCCCTCAAGAAAC
RINL Reverse Primer	GAGACTGGACGTGAATGGTGA
SOCS7 Forward Primer	GGGTCAAGACAGTCGGTGG
SOCS7 Reverse Primer	TCTCGGCCTCCGATTCCAA
TFDP2 Forward Primer	CTGCCTACCAATTCTGCTCAG
TFDP2 Reverse Primer	CGCTTCTGCTTTATCCGTTCT
PRKAA1 Forward Primer	TTGAAACCTGAAAATGTCCTGCT
PRKAA1 Reverse Primer	GGTGAGCCACAACTTGTTCTT
EIF4E Forward Primer	GAAACCACCCCTACTCCTAATCC
EIF4E Reverse Primer	AGAGTGCCCATCTGTTCTGTA
CBX5 Forward Primer	CTAGACAGGCGCGTGGTTAAG
CBX5 Reverse Primer	GCTCAGGGCAATCCAAGTTTT
SH3TC2 Forward Primer	TCGAGTGAGTGTATAGCCTCATC
SH3TC2 Reverse Primer	GGAGAGTGTCAGGTCTGGATT
PDE12 Forward Primer	GTAGTGCGCTGCGTACCTT
PDE12 Reverse Primer	GCGCTGCATGTTCTTGTGG
tRF-3009a-mimic	ACCCCACTCCTGGTACCA
tRF-3009a mut-mimic	AGGGGACTCCTGGTACCA
tRF-3001a-mimic	ATCCCACCGCTGCCACCA
tRF-3001a mut-mimic	AAGGGACCGCTGCCACCA
tRF-3003a-mimic	TCCGGGTGCCCCCTCCA
tRF-3003a mut-mimic	TGGCCGTGCCCCCTCCA

NC-mimic	miR1N0000001-1-5<br>from RiboBio (Guangzhou, China)

Abbreviations: NC, negative control.

Supplementary Figure

Figure S1. The pipeline of data preparation and SVM-GA model construction.

Figure S2. Cumulative distribution curve of the distance of seed sites to the 5' end, 3' end and nearest end. We observed that 3'-UTR sites immediately near the stop codon were less effective compared to sites elsewhere in the 3'-UTR. More sites within the remainder of the 3'-UTR tended to reside near the ends of the UTRs, especially the 5' end.

Figure S3. The correlation heatmap of 20 tRFs/miRNAs and mRNAs with the highest frequency among the target gene predictions.

Figure S4. Expression levels of predicted target genes were detected in MGC-803 cells after transfection with tRF mimics or mutated tRF mimics by qRT-PCR relative to the NC group.

Figure S1

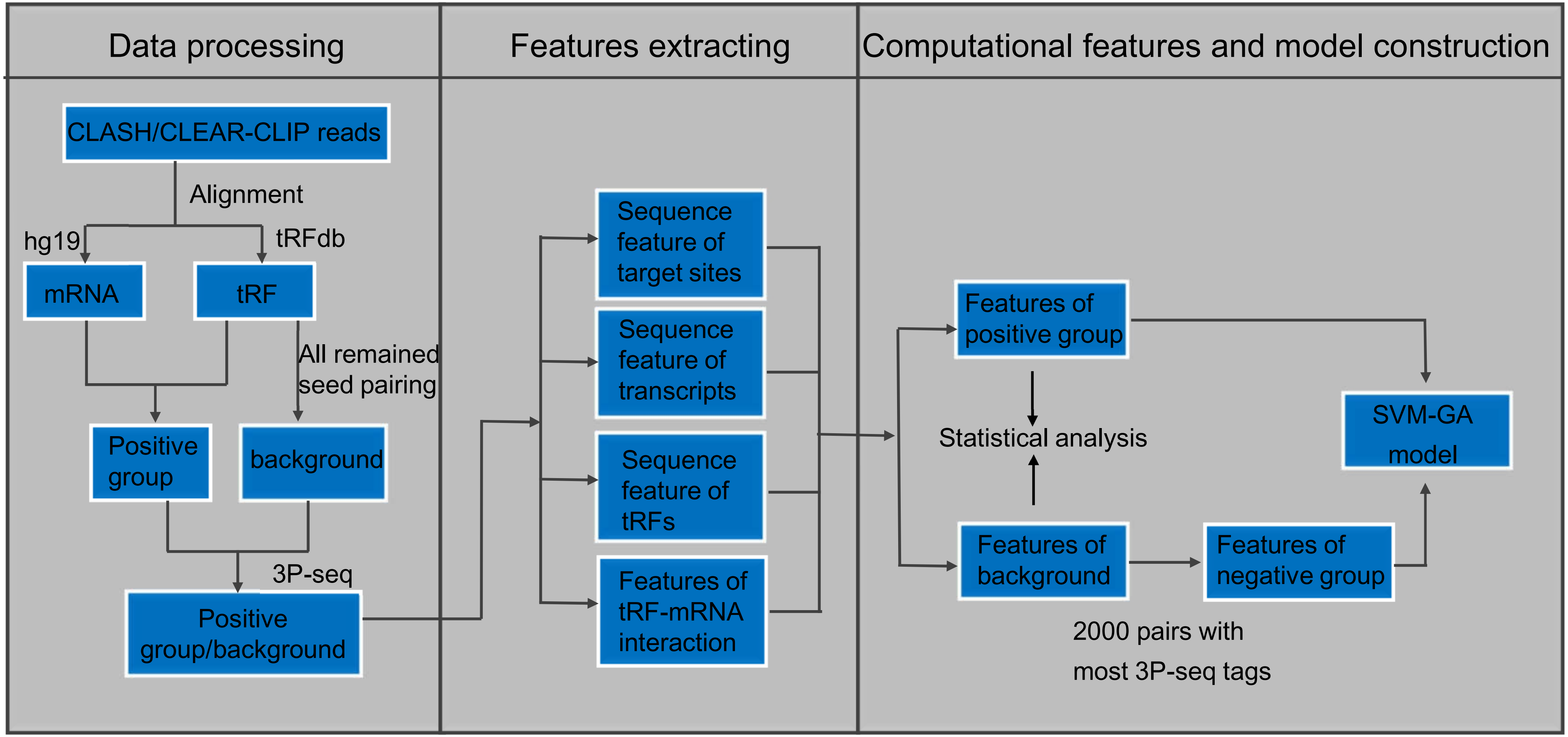
Data processing
Features extracting
Computational features and model construction
CLASH/CLEAR-CLIP reads
Alignment
hg19
tRFdb
mRNA
tRF
All remained seed pairing
Positive group
background
3P-seq
Positive group/background
Sequence feature of target sites
Sequence feature of transcripts
Sequence feature of tRFs
Features of tRF-mRNA interaction
Features of positive group
Statistical analysis
Features of background
Features of negative group
SVM-GA model
2000 pairs with most 3P-seq tags

Figure S2

Distribution of the distance of the seed match to the 5' end of 3'-UTR

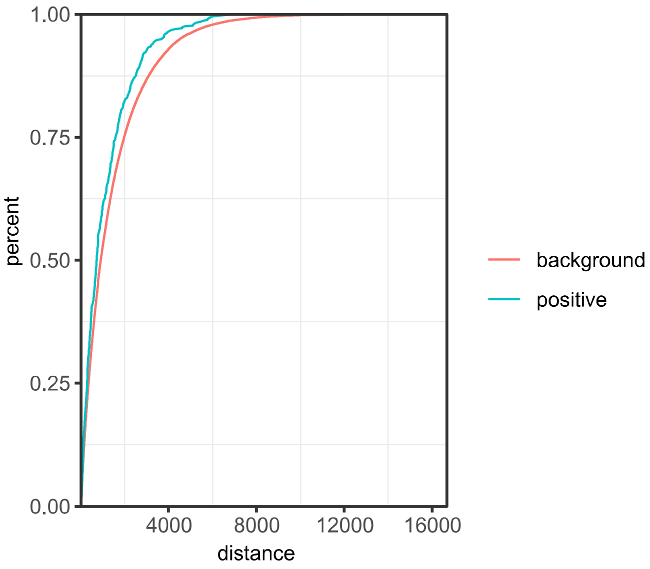

Distribution of the distance of the seed match to the 5' end of 3'-UTR

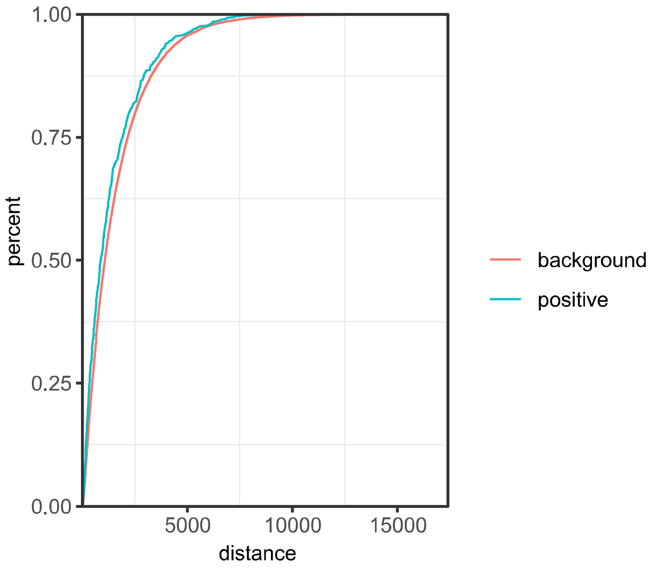

Distribution of the distance of the seed match to the nearest end of 3'-UTR

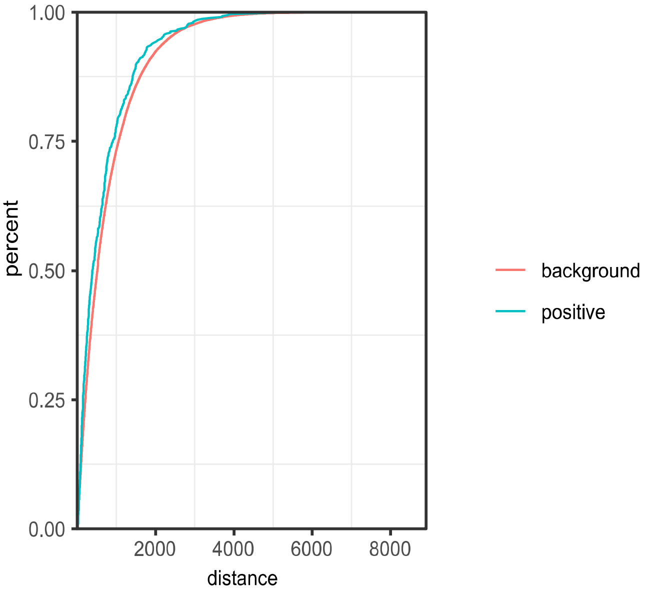

Figure S3

A

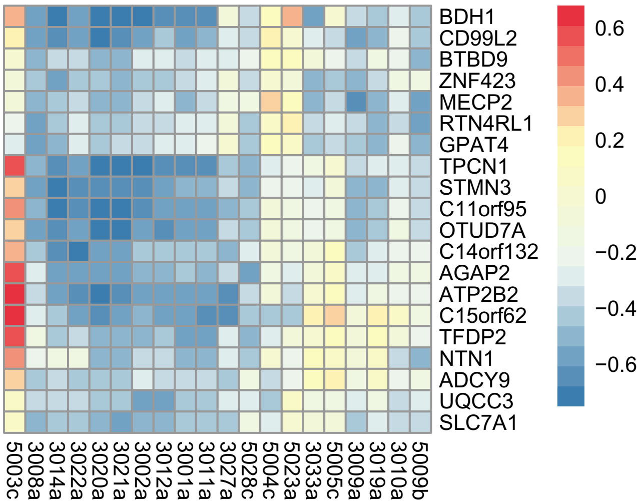

BDH1
CD99L2
BTBD9
ZNF423
MECP2
RTN4RL1
GPAT4
TPCN1
STMN3
C11orf95
OTUD7A
C14orf132
AGAP2
ATP2B2
C15orf62
TFDP2
NTN1
ADCY9
UQCC3
SLC7A1
0.6
0.4
0.2
0
−0.2
−0.4
−0.6
5003c
3008a
3014a
3022a
3020a
3021a
3002a
3012a
3001a
3011a
3027a
5028c
5004c
5023a
3033a
5005c
3009a
3019a
3010a
5009b

B

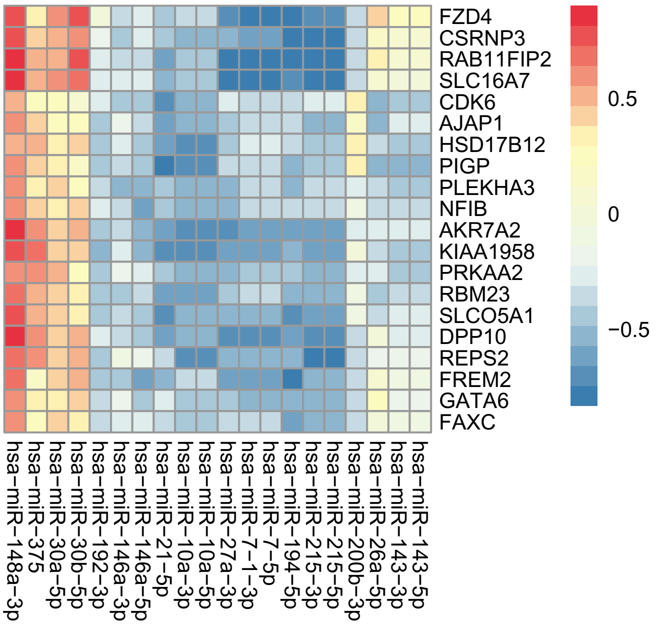

FZD4
CSRNP3
RAB11FIP2
SLC16A7
CDK6
AJAP1
HSD17B12
PIGP
PLEKHA3
NFIB
AKR7A2
KIAA1958
PRKAA2
RBM23
SLCO5A1
DPP10
REPS2
FREM2
GATA6
FAXC
0.5
0
−0.5
hsa−miR−148a−3p
hsa−miR−375
hsa−miR−30a−5p
hsa−miR−30b−5p
hsa−miR−192−3p
hsa−miR−146a−3p
hsa−miR−146a−5p
hsa−miR−21−5p
hsa−miR−10a−3p
hsa−miR−10a−5p
hsa−miR−27a−3p
hsa−miR−7−1−3p
hsa−miR−7−5p
hsa−miR−194−5p
hsa−miR−215−3p
hsa−miR−215−5p
hsa−miR−200b−3p
hsa−miR−26a−5p
hsa−miR−143−3p
hsa−miR−143−5p

Figure S4

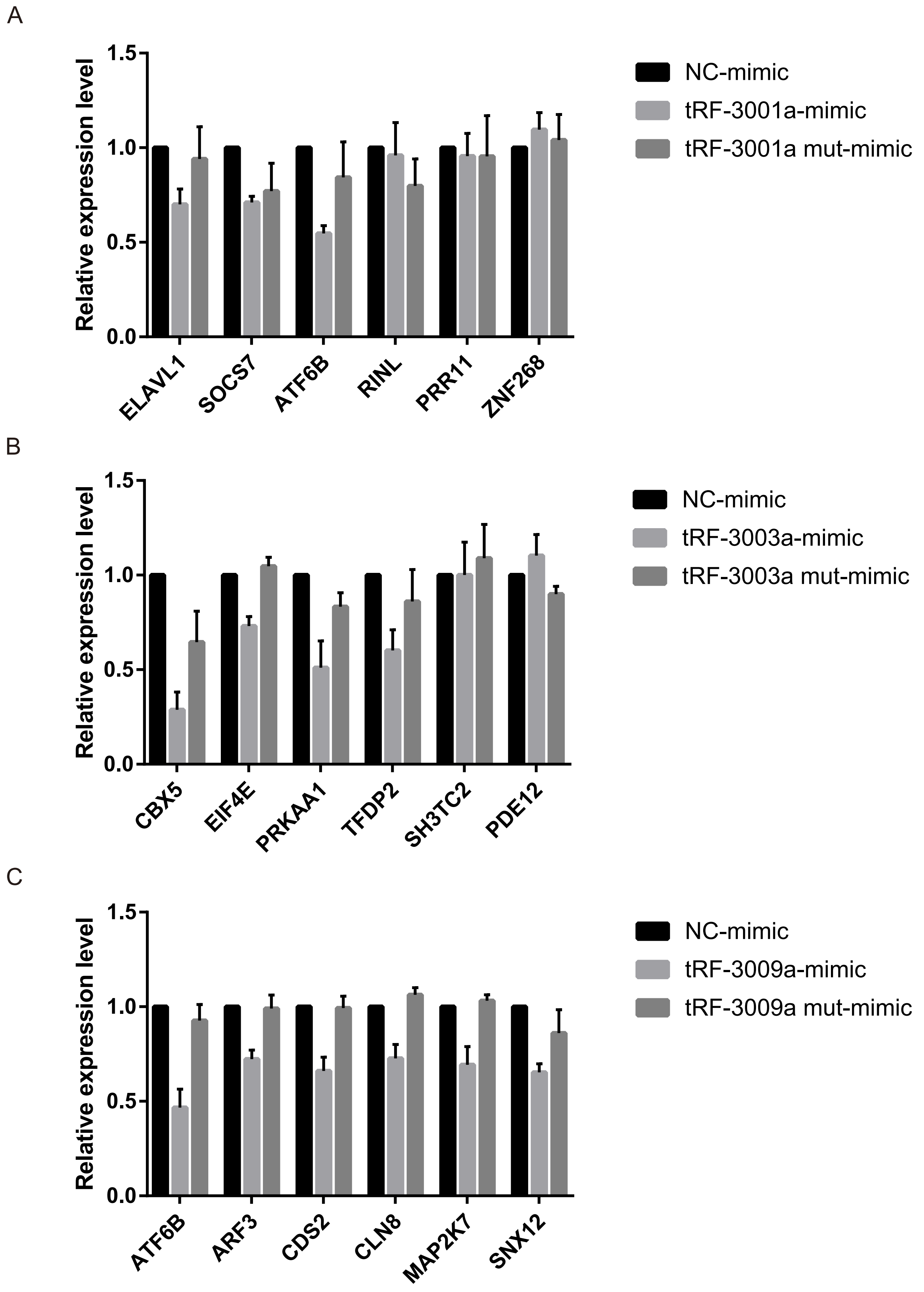

A
Relative expression level
0.0
0.5
1.0
1.5
ELAVL1
SOCS7
ATF6B
RINL
PRR11
ZNF268
NC-mimic
tRF-3001a-mimic
tRF-3001a mut-mimic
B
Relative expression level
0.0
0.5
1.0
1.5
CBX5
EIF4E
PRKAA1
TFDP2
SH3TC2
PDE12
NC-mimic
tRF-3003a-mimic
tRF-3003a mut-mimic
C
Relative expression level
0.0
0.5
1.0
1.5
ATF6B
ARF3
CDS2
CLN8
MAP2K7
SNX12
NC-mimic
tRF-3009a-mimic
tRF-3009a mut-mimic
